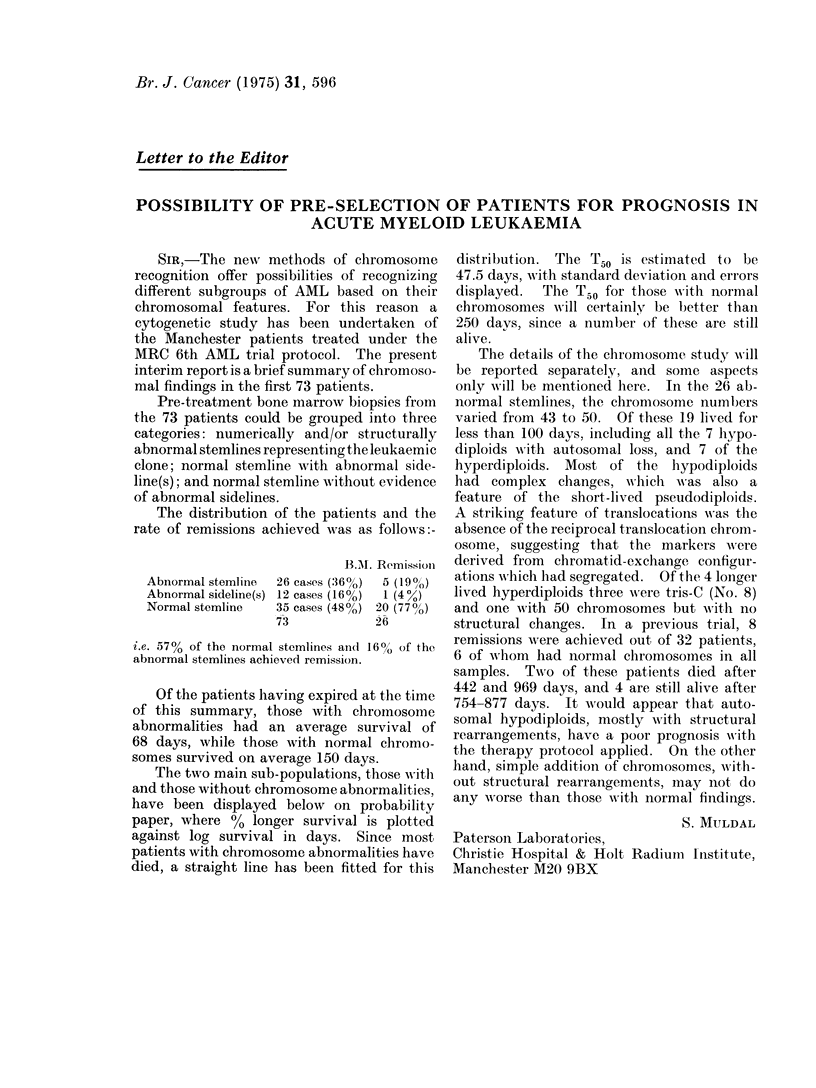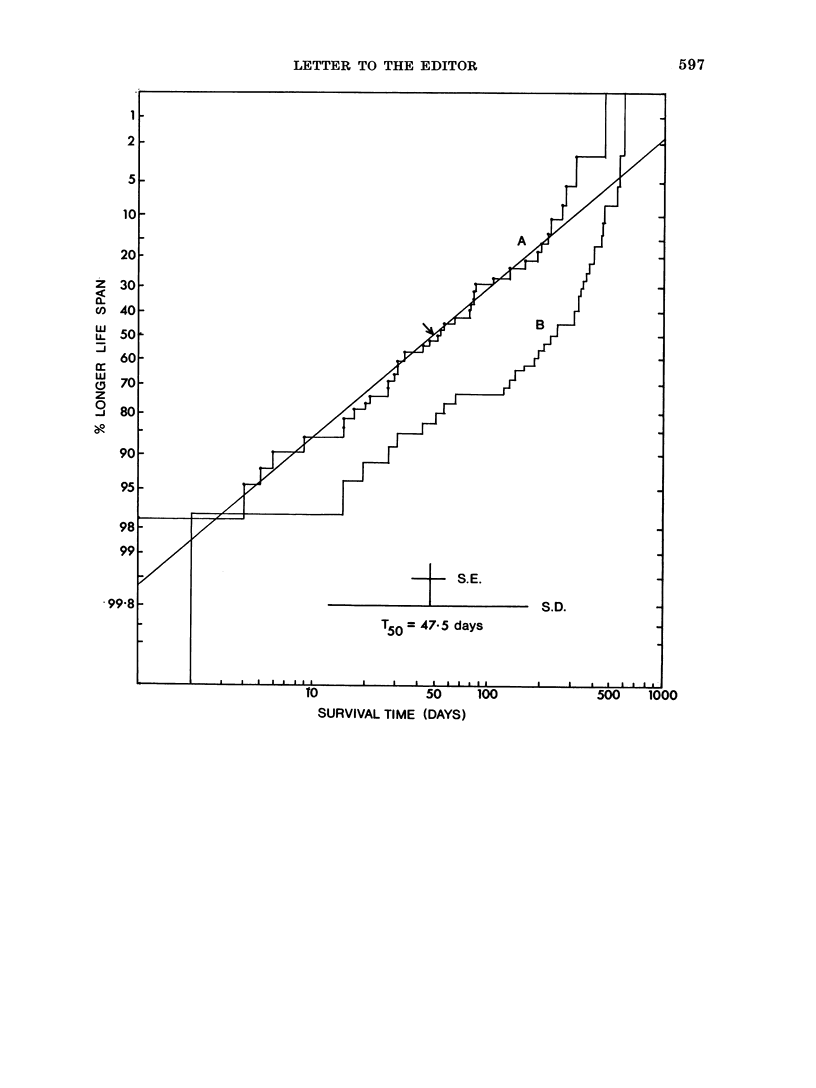# Letter: Possibility of pre-selection of patients for prognosis in acute leukaemia.

**DOI:** 10.1038/bjc.1975.103

**Published:** 1975-05

**Authors:** S. Muldal


*Br. J. Cancer* (1975) **31**, 596

### *Letter to the Editor*

### POSSIBILITY OF PRE-SELECTION OF PATIENTS FOR PROGNOSIS IN ACUTE MYELOID LEUKAEMIA

SIR,—The new methods of chromosome recognition offer possibilities of recognizing different subgroups of AML based on their chromosomal features. For this reason a cytogenetic study has been undertaken of the Manchester patients treated under the MRC 6th AML trial protocol. The present interim report is a brief summary of chromosomal findings in the first 73 patients.

Pre-treatment bone marrow biopsies from the 73 patients could be grouped into three categories: numerically and/or structurally abnormal stemlines representing the leukaemic clone; normal stemline with abnormal side-line(s); and normal stemline without evidence of abnormal sidelines.

The distribution of the patients and the rate of remissions achieved was as follows:-

| | | B.M. Remission |
|---|---|---|
| Abnormal stemline | 26 cases (36%) | 5 (19%) |
| Abnormal sideline(s) | 12 cases (16%) | 1 (4%) |
| Normal stemline | 35 cases (48%) | 20 (77%) |
| | 73 | 26 |

*i.e.* 57% of the normal stemlines and 16% of the abnormal stemlines achieved remission.

Of the patients having expired at the time of this summary, those with chromosome abnormalities had an average survival of 68 days, while those with normal chromosomes survived on average 150 days.

The two main sub-populations, those with and those without chromosome abnormalities, have been displayed below on probability paper, where % longer survival is plotted against log survival in days. Since most patients with chromosome abnormalities have died, a straight line has been fitted for this distribution. The $T_{50}$ is estimated to be 47.5 days, with standard deviation and errors displayed. The $T_{50}$ for those with normal chromosomes will certainly be better than 250 days, since a number of these are still alive.

The details of the chromosome study will be reported separately, and some aspects only will be mentioned here. In the 26 abnormal stemlines, the chromosome numbers varied from 43 to 50. Of these 19 lived for less than 100 days, including all the 7 hypodiploids with autosomal loss, and 7 of the hyperdiploids. Most of the hypodiploids had complex changes, which was also a feature of the short-lived pseudodiploids. A striking feature of translocations was the absence of the reciprocal translocation chromosome, suggesting that the markers were derived from chromatid-exchange configurations which had segregated. Of the 4 longer lived hyperdiploids three were tris-C (No. 8) and one with 50 chromosomes but with no structural changes. In a previous trial, 8 remissions were achieved out of 32 patients, 6 of whom had normal chromosomes in all samples. Two of these patients died after 442 and 969 days, and 4 are still alive after 754–877 days. It would appear that autosomal hypodiploids, mostly with structural rearrangements, have a poor prognosis with the therapy protocol applied. On the other hand, simple addition of chromosomes, without structural rearrangements, may not do any worse than those with normal findings.

S. MULDAL

Paterson Laboratories,
Christie Hospital & Holt Radium Institute,
Manchester M20 9BX